# A rare case of acute eosinophilic pneumonia induced by vaping-associated lung injury: a case report

**DOI:** 10.1186/s12890-023-02581-7

**Published:** 2023-08-09

**Authors:** Abir Hamad Alsaid, Alaeldin Elfaki, Moath Thamer Alkhouzaie, Raghad Abdullah Alghamdi

**Affiliations:** 1 Department of Internal Medicine, King Fahd Hospital of the University, Khobar, Saudi Arabia; 2 Department of Medicine and Surgery, Imam Abdulrahman Bin Faisal University, Dammam, Saudi Arabia

**Keywords:** Acute eosinophilic pneumonia (AEP), Electronic cigarette or vaping product use-associated lung injury (EVALI), Eosinophilic lung disease (ELD), Eosinophils, Vaping

## Abstract

**Background:**

Acute eosinophilic pneumonia (AEP) is well-known as one of the primary eosinophilic pulmonary diseases of unknown etiology. It’s defined as a febrile illness along with acute onset respiratory failure that is commonly misdiagnosed at the initial presentation as infectious pneumonia. Despite the fact that AEP sometimes classified as idiopathic as no exact cause can be identified in most cases, it has been suggested recently to be linked with electronic cigarette or vaping products and associated with electronic cigarette or vaping associated lung injury (EVALI). Therefore, history of recent tobacco smoking or vaping exposure along with peripheral eosinophilia are crucial clinical findings suggestive of AEP.

**Case presentation:**

A previously healthy 17-year-old female presented to the Emergency Room with one day history of progressively worsening shortness of breath accompanied by left sided pleuritic chest pain and fever. She wasn’t taking any medications, denied traditional cigarette smoking, exposure to pulmonary irritants, recent travel and had no history of close contact with sick patient. She recently started vaping 20 days prior to the presentation. Initially, she was admitted with a presumptive diagnosis of atypical pneumonia but was found to have AEP due to a recent vaping exposure.

**Conclusion:**

Vaping is a well-known health hazard that has become a growing trend among adolescents and have been promoted as a safe and effective alternative to traditional cigarettes. The etiology of AEP remains unclear, but many studies suggest a possible link with recent tobacco smoking or vaping. A key challenge for this clinical entity is to reach the diagnosis after excluding all other pulmonary eosinophilia causes, and it has an excellent prognosis if diagnosed early and treated appropriately.

## Established Facts

• The eosinophilic lung diseases (ELD) are a diverse group of pulmonary conditions associated with peripheral or tissue eosinophilia.
• Acute eosinophilic pneumonia (AEP) is an extremely rare disorder characterized by nonproductive cough, dyspnea, and fever that typically present in less than four weeks.
• The cause of AEP is unknown, but several factors are necessary for the development of the disorder, with association of a triggering factor in a predisposed individual.

**Novel Insights**

• The association of AEP with recent tobacco smoking or vaping exposure suggests a possible link.
• The treatment of AEP depends on the underlying cause when recognized, but glucocorticoids are the treatment of choice when it is caused by noninfectious causes.
• Prognosis of AEP is excellent if treated appropriately with corticosteroids.

## Introduction

The eosinophilic lung diseases (ELD) represent a heterogeneous group of pulmonary conditions, which manifest by the presence of peripheral blood eosinophilia in the absence of other causes [1]. Although eosinophils are found normally with few numbers (less than 2%) on bronchoalveolar lavage (BAL), it is expected to be higher in case of ELD [1]. Acute eosinophilic pneumonia (AEP) known as a febrile illness associated with acute onset respiratory failure that is commonly misdiagnosed in the initial presentation as infectious pneumonia. The cause of AEP is most commonly unknown, but identifiable causes include smoking, inhalational exposures, medications, and infections have all been linked to a higher acquisition rate of the disease. Inflammation occurs through excessive activation of type-2 immune cells and release of type-2 cytokines such as interleukin-5 (IL-5). This results in eventual inflammatory cascade with excessive eosinophil recruitment to the lung parenchyma [2, 3]. The treatment of AEP depends on the underlying cause when recognized, but glucocorticoids are the treatment of choice when AEP is caused by noninfectious causes [1, 2].

## Case report

**Fig. 1:**
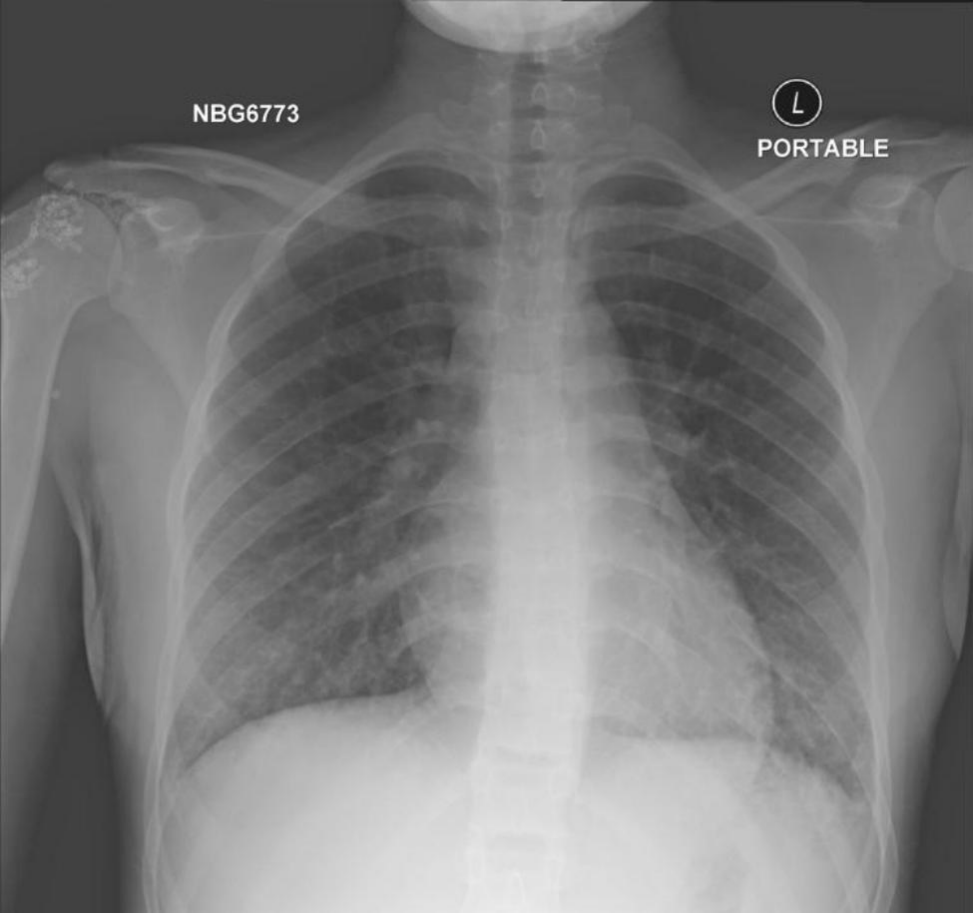
Chest x-ray showing bilateral lower lung zones airspace opacities with subtle left-sided pleural effusion

**Fig. 2:**
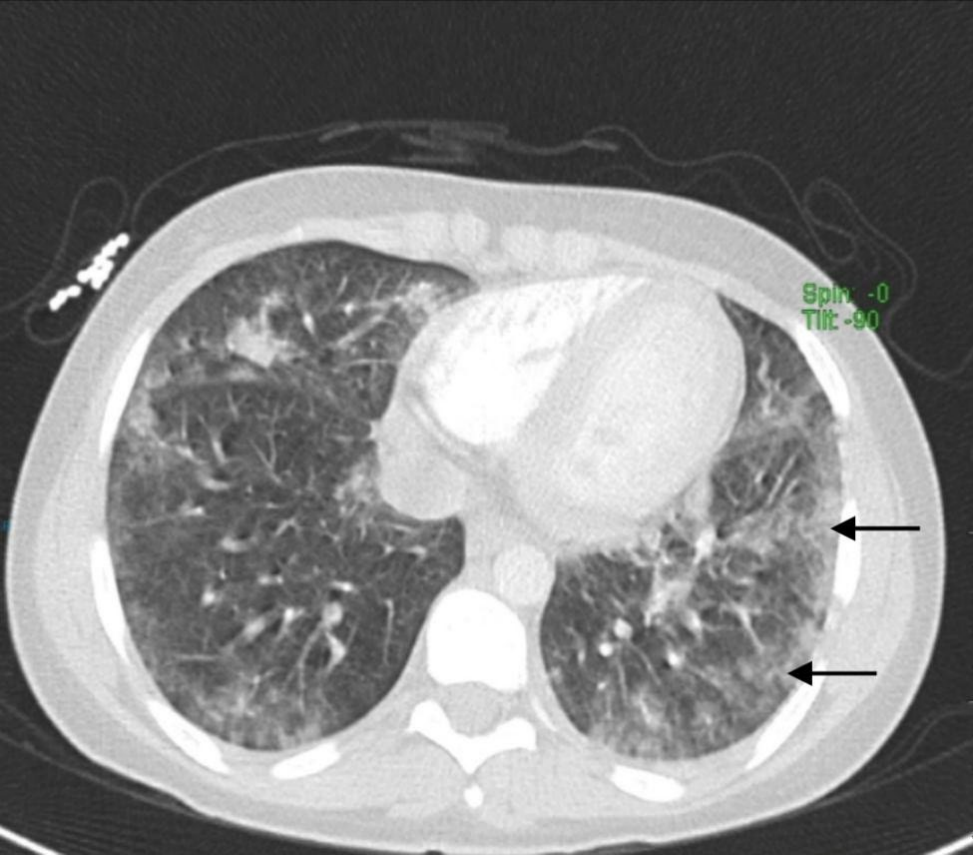
CT with contrast showing multiple scattered peripheral ground-glass consolidation suggestive of an inflammatory process

A 17-year-old female developed a sudden onset shortness of breath for one-day duration associated with pleuritic left sided chest pain and fever. She denied prior respiratory infections and systematic review was unremarkable. She wasn’t taking any medications neither prescribed nor over the counter, denied recent travel and had no history of close contact with sick patient. She recently started vaping 20 days prior to the presentation. There was no significant past medical or surgical history. Family history was significant only for atopy. Initial vital signs showed temperature: 39.5 °C, heart rate (HR): 111 beats per minute (BPM), respiratory rate (RR): 23 breaths per minute, blood pressure (BP): 110/72 millimeters of mercury (mmHg), oxygen saturation (SpO2): 96% on room air. There was neither accessory muscle use nor chest wall tenderness, and her chest examination revealed good equal air entry bilaterally, normal vesicular breathing and bilateral basal crepitations. Laboratory diagnosis was notable for a WBC count of 11.5 × 10^3^/µL, with a normal differential count except for 14% eosinophils; inflammatory markers revealed a CRP: 3.5 mg/dL, and ESR: 6 mm/hr. Chest x-ray (CXR) showed bilateral lower lung zones airspace opacities with subtle left-sided pleural effusion (shown in Fig. 1). As the patient was tachypneic with progressive hypoxia, Computed Tomography Angiography (CTA) was done to rule out pulmonary embolism (PE), which showed multiple scattered peripheral ground-glass consolidation suggestive of an inflammatory process (shown in Fig. 2). Three COVID-19 PCR tests came to be negative, thus COVID-19 infection was excluded. A course of antibiotics including Intravenous (IV) Azithromycin 500 mg and IV ceftriaxone 1 g was started, but the symptoms failed to improve and progressed to hypoxemic respiratory failure (pH 7.36, PaCO2: 47.2 mmHg, PaO2: 29.1: mmHg) that required high flow oxygen. Flexible bronchoscopy with bilateral BAL was performed revealing high eosinophils count 84% with no other infectious etiologies detected. Given her acute onset of symptoms, negative alternative workup, and significant BAL eosinophilia the diagnosis of AEP was made. Therefore, oral steroid was prescribed in form of prednisone 40 mg/day for two weeks then taper by 5 mg every week. Three weeks after discharge, the patient came back for follow up. Her symptoms improved and chest high resolution computed tomography scan (HRCT) showed interval resolution of the bilateral opacity which is consistent with the treatment response.

## Discussion

AEP is a rare and a serious condition characterized by nonproductive cough, dyspnea, and fever that is typically present in less than four weeks [4]. Currently, the modified Philit criteria is used to diagnose AEP (Table 1), it includes having an acute respiratory illness for less than or equal to one month duration, pulmonary infiltrates seen in CXR or computed tomography (CT), more than 25% eosinophils in BAL fluid and absence of other known causes of pulmonary eosinophilia [2]. These criteria were entirely observed in our patient with BAL of 84% eosinophils. Patients typically present with normal peripheral blood eosinophil level; however, it can rise afterwards [5]. This was the case in our patient, at the initial presentation the peripheral blood eosinophil level was 2.3%, and after four days it raised up to 14.5%. Differential diagnosis for AEP includes eosinophilic granulomatosis with polyangiitis, hypereosinophilic syndrome, allergic bronchopulmonary aspergillosis [2], pulmonary fungal and parasitic infections, and medications induced [5]. As it crucial to consider all these differential diagnoses, it was ruled out in our patient before confirming the diagnosis of AEP.

**Table 1:** Modified Philit criteria to diagnose acute eosinophilic pneumonia (AEP) [5]:

Modified Philit criteria	
■ ■ ■ ■	Acute respiratory illness ≤ 1-month duration. Pulmonary infiltrates on CXR or CT. > 25% eosinophilia in BAL fluid or eosinophilic pneumonia on lung biopsy. Absence of other causes of pulmonary eosinophilia.

While the disease can be idiopathic, a well-known entity of AEP occurs following exposure to new inhalants, mostly in first-time cigarette smokers [6, 7], firefighters exposed to high concentrations of World Trade Center dust [8], military service personnel in the Middle East [9], and lately, in vape users [6, 10]. Our patient denied encountering any of these exposures, except a recent start of vaping, which makes it the most likely causative agent. The mechanism by which e-cigarette smoking or vaping induces AEP is suspected to be a strong inflammatory stimulus that recruits macrophages and neutrophils to lung tissue, which may be causing eosinophil-rich exudate within the alveolar spaces [6]. A dramatic increase in the cases of pulmonary toxicity associated with vaping was recognized in 2019, and the term e-cigarette or vaping use-associated lung injury (EVALI) was given by the Centers for Disease Control and Prevention (CDC) [11]. EVALI has a wide range of clinical manifestations and AEP has been described as one of them, however it remains extremely rare.

Previous studies have reported a close relationship between vaping exposure or use of e-cigarette and AEP. In 2019, Zhaohui L. Arter and colleagues reported a case of AEP in young female after a recent start of vaping. Initially her presentation was consistence with pneumonia, in which she received a course of antibiotic with no improvement, and she developed moderate respiratory distress and hypoxemia. BAL was performed, revealing 26% eosinophils. After confirming the diagnosis of AEP and the patient started to show improvement, she was discharged home with oral prednisone 60 mg/day with subsequent tapering. Similarly was done in our patient, however prednisone 40 mg/day was initiated, and the patient showed a dramatic response with no need to consider further dose adjustment [6]. Although most cases of AEP are reported in the male population, further research is required to clarify whether AEP is more common in males due to the confounding factors such as smoking prevalence, job associated irritants exposure, or is there any link to the organic factors [6].

Additionally, two other cases of AEP linked to the vaping exposure have been published in 2020. Puebla Neira et al. report a case of AEP linked to recent use of counterfeit tetrahydrocannabinol oil vaping in a young male with similar initial presentation to our patient. He was discharged on prednisone 40 mg/day with tapering dose. On follow up, patient had complete resolve of the symptoms with a normal physical exam [4]. Another case of vaping induces AEP was reported in a young female after a recent use of the electronic vape of tetrahydrocannabinol (THC) substance [5]. Due to the wide variety of vaping devices and the different type of substances, more research is needed to determine the exact cause of e-cigarette induced AEP.

Management of AEP involves administration of high-dose systemic glucocorticoids intravenously until the respiratory failure resolves, followed by an oral steroid taper. Although the ideal duration of a steroid taper has not been identified, it is generally recommended that a slow taper extending at least two weeks after radiographic and laboratory improvements have been demonstrated [1, 2].

## Conclusion

Vaping is a well-known health hazard that has become a growing trend among adolescents and have been promoted as a safe and effective alternative to traditional cigarettes. The etiology of AEP remains unclear, but many studies suggest a possible link with recent tobacco smoking or vaping exposure. Thus, physicians should consider AEP in a previously healthy patients with hypoxic respiratory failure who have a history of recent tobacco smoking or vaping exposure along with peripheral eosinophilia. In this case of clear association, our patient had no other traditional exposures which could cause AEP, leaving a recent start of vaping use as the most likely triggered factor. The challenge lies in the timely diagnosis of this clinical entity, since its frequently resemble an infectious process with chest radiographs mimicking bacterial pneumonia at the initial presentation. Patients with AEP have a rapid and excellent prognosis if diagnosed and treated appropriately.

## Data Availability

The authors confirm that the data supporting the findings of this study are available within the article.
